# Rice Coconut Yogurt: Insights Into Physicochemical Properties, Microbial Stability, and Consumer Acceptance as a Plant‐Based Alternative

**DOI:** 10.1002/fsn3.71466

**Published:** 2026-01-16

**Authors:** Md. Naimur Rahman, Md. Nahidul Islam, Md. Sazzat Hossain Sarker, Md. Sultan Mahomud

**Affiliations:** ^1^ Department of Nutrition and Food Engineering Daffodil International University Dhaka Bangladesh; ^2^ Department of Food Engineering Gazipur Agricultural University Gazipur Bangladesh; ^3^ Institute of Food Safety and Processing Gazipur Agricultural University Gazipur Bangladesh; ^4^ Department of Food Engineering and Technology Hajee Mohammad Danesh Science and Technology University Dinajpur Bangladesh

**Keywords:** bacterial count, coconut milk, microstructure, plant‐based alternative, rice milk, sensory, viscosity, water holding capacity

## Abstract

The rising consumer demand for plant‐based and non‐dairy yogurts prompted this investigation into the potential of incorporating coconut milk and rice milk into yogurt production. Yogurt samples were prepared by blending skim milk powder (SMP) with rice milk, coconut milk, cow milk, and a 50:50 rice‐coconut milk mixture, alongside a control made from 100% cow milk. Comprehensive analyses evaluated physicochemical properties—pH, acidity, water‐holding capacity, syneresis, viscosity, total soluble solids, moisture content, total solids, and color—as well as microbiological counts and sensory attributes. Results revealed that coconut milk yogurt had the lowest pH (4.10 ± 0.021) and highest acidity (1097.67 ± 7.51 mg/100 mL), while adding SMP to cow milk increased pH to 5.02 ± 0.021 and reduced acidity. Coconut milk yogurt also exhibited superior color, water‐holding capacity (57.46% ± 0.174%), and viscosity, whereas rice milk yogurt showed higher syneresis (19.43% ± 0.404%) but acceptable microbiological and sensory profiles. Notably, rice milk yogurt gained the highest consumer acceptability, surpassing cow and coconut milk variants. Overall, the findings suggest that coconut milk yogurt is a promising dairy alternative, particularly for individuals with lactose intolerance and dairy allergies, with rice milk yogurt also representing a viable, nutritionally beneficial option.

## Graphical abstract

This study demonstrates that rice and coconut milk can serve as effective bases for nutrient‐rich, plant‐based yogurts with properties similar to those of dairy yogurt. Coconut milk yogurt exhibited superior stability and acidity, while rice milk yogurt offered high consumer acceptability. These findings suggest that coconut milk yogurt is a promising dairy alternative, particularly for individuals with lactose intolerance and dairy allergies, with rice milk yogurt also representing a viable, nutritionally beneficial option.

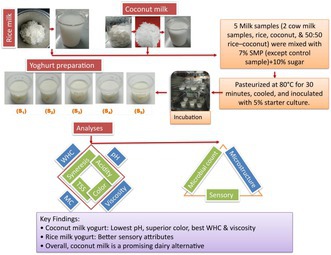

## 1 Introduction

Yogurt is a widely consumed fermented milk product appreciated globally for its nutritional benefits (Nandakumar et al. 2022). It is traditionally produced by fermenting milk with live bacteria, primarily *Lactobacillus bulgaricus* and *Streptococcus thermophilus* , which convert milk sugars into acid, creating its characteristic texture (Hadjimbei et al. 2022). Yogurt is rich in calcium, proteins, and bioactive compounds, and serves as an excellent vehicle for probiotics that promote gut health (Montemurro et al. 2021; Rasika et al. 2021). Its diverse formulations depend on the strains of bacteria used, flavorings, and the type of milk—be it plant or animal‐based (Belewu et al. 2013).

In recent years, consumer demand for plant‐based yogurts—such as those made from soy, coconut, oat, and almond—has surged. This shift is driven by health concerns, including lactose intolerance and allergies to animal proteins, as well as environmental sustainability and vegetarianism (Amin et al. 2025). Although bacteria can degrade lactose to produce lactic acid, individuals with lactose sensitivity or allergies may experience adverse reactions, and plant‐based alternatives have gained popularity because they lack lactose and casein (Priya 2016; Rasika et al. 2021).

Coconut, a staple food on many islands, is high in fats, energy, and essential nutrients, making coconut milk (extracted from grated coconut meat) a valuable ingredient across food industries (Adelekan and Alamu 2021; Belewu and Abodunrin 2007). It is also recognized for its health benefits, and coconut milk yogurt has been identified as a nutritious and palatable product (Imele and Atemnkeng 2001). Conversely, rice is a gluten‐free, hypoallergenic grain that provides essential nutrients and is a staple for half of the world's population (Ito and Lacerda 2019; Roy et al. 2025). Rice milk is a low‐calorie, low‐fat alternative rich in vitamins B12, D, A, and calcium, making it suitable for those with lactose intolerance (Belewu et al. 2013; Fawzi et al. 2022).

Extensive research has focused on plant‐based milk substitutes and their fermentation into yogurt, with evidence suggesting improvements in nutritional quality, vitamin synthesis, and digestibility (Rasika et al. 2021). However, certain plant sources, such as soy and nuts, pose allergen risks, whereas coconut allergies are relatively rare (Kamal et al. 2025; Rahman et al. 2026). Despite existing research on plant‐based yogurt formulations, a significant research gap remains regarding the comparative analysis of rice milk, coconut milk, and their combination in yogurt production, particularly concerning their physicochemical, microstructural, and sensory properties over storage. Previous studies often focus on single plant‐based milk types (Belewu et al. 2013) and rarely perform a comprehensive evaluation that includes microstructure, stability, and consumer acceptability in parallel. This study uniquely contributes by comparing rice milk, coconut milk, and their 50:50 blend with traditional cow milk yogurt, providing new insights into their potential as nutritious, allergen‐free, and sustainable dairy alternatives. Challenges in developing rice‐ and coconut‐based yogurts include achieving suitable texture and firmness, often requiring the addition of skim milk powder (SMP) to increase solid content. Additionally, the high fat content in coconut milk can affect surface hardness during refrigeration.

The present study aims to fill this gap by evaluating the physicochemical, microbiological, microstructural, and sensory characteristics of yogurt made from rice and coconut milk. To our knowledge, this is among the first comprehensive attempts to compare these plant‐based yogurts with traditional cow milk yogurt, supporting their development as nutritious, allergen‐free, and sustainable dairy alternatives. This research could open new avenues for valorizing rice and coconut milk in probiotic food formulations, catering to the growing demand for plant‐based functional foods.

## 2 Materials and Methods

### 2.1 Raw Materials Collection

Rice, fresh coconuts, skim milk powder, and sugar were purchased from a local market in Salna, Gazipur. Bacterial cultures ( *Lactobacillus bulgaricus* and *Streptococcus thermophilus* ) were sourced from Christian Hansen (Boege Alle 10‐12, 2970 Hoersholm, Denmark). Additional equipment was utilized from the laboratory stock at the Department of Food Engineering, Gazipur Agricultural University, Gazipur, Bangladesh.

The selection of coconut and rice milk was based on their hypoallergenic nature, nutritional profiles, and growing consumer demand for plant‐based, gluten‐free, and sustainable dairy alternatives. Coconut milk is known for its rich lipid content, which contributes to a desirable texture and flavor in yogurt. Meanwhile, rice milk offers a low‐cost, nutritious, and hypoallergenic option suitable for individuals with lactose intolerance and gluten sensitivity.

For the preparation of rice milk, BR29 rice ( *Oryza sativa* ) was used due to its widespread availability and its lower amylose content, which tends to produce a smoother, more palatable milk with favorable functional and sensory properties. This choice was supported by literature suggesting that long‐grain rice yields rice milk with desirable viscosity and consistency for fermentation processes.

Rice milk was prepared following the method by Fawzi et al. (2022) with minor modifications. Initially, the rice was washed three times with tap water. Two cups of rice were then soaked in hot water (70°C–80°C) for 2 h until soft enough to break in half with a fingernail but still uncooked. The soaked rice was blended with four cups of water for around 1 min, avoiding complete pulverization. The mixture was then filtered through a double‐layer cheesecloth, and the rice milk was stored chilled until required.

**FIGURE 1:**
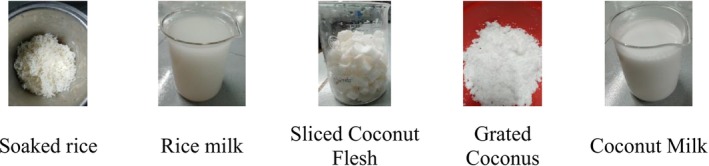
Various steps of rice and coconut milk preparation.

Coconut milk was made based on the procedure by Belewu and Abodunrin (2007), with slight adjustments. The coconut was shelled, and the meat was removed with a blunt knife. After peeling off the brown skin and washing the coconut meat, it was grated and soaked in warm water for 45 min. The mixture was pressed through a 0.18 mm sieve to obtain an opaque, sweet‐tasting coconut milk. Various steps of rice coconut milk production are illustrated in Figure 1.

According to Rahman et al. (2024), the culture was prepared by dissolving 12 g of skim milk powder in 100 mL of water, heating it to 80°C for 15 min, and then cooling it to 45°C–50°C. Freeze‐dried yogurt lactic starter cultures of *Lactobacillus bulgaricus* and *Streptococcus thermophilus* were added at a rate of 7 mg per 100 mL of skim milk. The mixture was incubated at 42°C for at least 6 h, then refrigerated at 4°C until further use.

### 2.2 Preparation of Rice Milk and Coconut Milk Yogurt Samples

**FIGURE 2:**
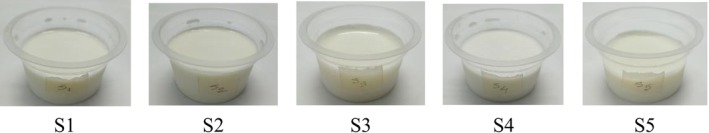
Pictorial view of the prepared yogurt samples. Where, S1, cow milk (control); S2, cow milk + SMP; S3, rice milk + SMP; S4, coconut milk + SMP; S5, rice coconut blend + SMP.

Yogurt samples were prepared following the method of Belewu et al. (2013), with some modifications. Varying amounts of rice milk, coconut milk, and skim milk powder (SMP) were used, along with a control sample. Specifically, five beakers were prepared: beaker 1 contained cow milk (control), beaker 2 contained normal milk with 7% SMP, beaker 3 contained rice milk with 7% SMP, beaker 4 contained coconut milk with 7% SMP, and beaker 5 contained a mixture of 50% rice milk and 50% coconut milk with 7% SMP (Figure 2). This addition served dual purposes: to standardize the TSS across all samples and to provide a rich source of milk proteins, thereby enhancing fermentation efficiency, texture, and probiotic viability. The consistent application of SMP across all treatments was essential for ensuring reproducibility and accurate interpretation of comparative results. All components were pasteurized at 80°C for 30 min using indirect heating. This step was essential to gelatinize the starch in the plant‐based milks, improve viscosity, and reduce endogenous microorganisms (Baskar et al. 2022). During pasteurization, 10% sugar was added to each beaker. Afterward, the mixtures were cooled to 45°C–50°C and inoculated with 5% of the yogurt culture. The inoculated mixtures were then transferred into 80 g plastic containers and incubated at 42°C for 8–10 h. Following incubation, the yogurt samples were stored at 4°C, and analyses were performed on days 1 and 7 of storage.

### 2.3 Determination of pH

The pH changes in the yogurt samples were determined throughout the storage period in accordance with Anjum et al. (2025). A laboratory pH meter equipped with a glass electrode (HANNA, Instrument, Portugal) was used for measurements. The pH was measured at intervals of 1 day and 7 days after storage by directly immersing the electrode into the yogurt samples. The pH meter was calibrated using standard buffers of pH 7 and pH 4 prior to measurements.

### 2.4 Determination of Acidity of the Yogurt

Using the method described by Kamal‐Eldin et al. (2020), the total titratable acidity was calculated and expressed as a percentage of lactic acid. The determination involved mixing 10 g of yogurt with 20 mL of distilled water, then titrating the mixture with 0.1 N NaOH while using phenolphthalein as an indicator until a light pink endpoint was reached. The lactic acid content (mg/100 mL of yogurt) was calculated using the formula:

$$\text{Lactic acid}\frac{\mathrm{mg}}{100}\mathrm{mL}\;\text{of}\;m\mathrm{ilk}=\frac{\mathrm{Vg}\times N\times 90\times 100}{\mathrm{Vm}}$$

where *Vg* was the volume of NaOH solution used, *N* was the concentration of the NaOH solution, 90 was the equivalent weight of lactic acid, and *Vm* was the volume of yogurt used for titration.

### 2.5 Determination of Water Holding Capacity

With a few minor adjustments, the water holding capacity (WHC) of the yogurt samples was calculated following the technique described by Rahman et al. (2024). A 15 g sample of yogurt was placed in a centrifuge tube and spun at 8000 × g for 15 min at 4°C. The supernatant whey was then discarded, and the separated whey was weighed. The WHC was determined as a percentage using the formula:

$$\mathrm{WHC}\%=\left(1-\frac{W1}{W2}\;\right)\times 100$$

where W_1_ is the weight of the whey obtained after centrifugation, and W_2_ is the initial weight of the yogurt sample.

### 2.6 Determination of Susceptibility to Syneresis

The susceptibility to syneresis (STS) of the yogurt samples was determined according to the method described by Mahomud et al. (2024). A 20 mL sample of yogurt was placed on filter paper and positioned over a funnel to facilitate whey drainage. After 3 h, the volume of whey collected in a beaker was measured and used as the syneresis index. The STS was calculated using the formula:

$$\mathrm{STS}\;\left(\%\right)=\;\frac{V1}{V2}\;\times 100$$

where V_1_ was the volume of whey collected after drainage, and V_2_ was the initial volume of the yogurt sample.

### 2.7 Determination of TSS

Prior to the addition of sugar and fermentation, the total soluble solids (TSS) of each milk suspension (coconut milk, rice milk, their 50:50 blend, and control milk) were measured using a refractometer (Hanna HI96801) at 21°C. This step ensured the initial soluble solids content was documented and allowed for standardization across formulations. Normal cow milk showed TSS of 10 °Brix, rice milk 10.5 °Brix, coconut milk 9.2 °Brix, rice and coconut milk blend 9.5 °Brix. Later on, each yogurt sample's TSS content was calculated using the standard procedures (Bapary et al. 2024). TSS of the yogurt samples was measured using the same refractometer under the same conditions.

### 2.8 Determination of Viscosity

A slight adjustment was made to the viscosity measurement procedure based on the approach described by Dinkci et al. (2021). A viscometer (MYR VR‐3000), equipped with the appropriate spindle (Spindle no. 3) and set at 20 rpm, was used to measure the apparent viscosity of the yogurt samples after stirring for 30 s. The shear was maintained for 30 s, and the results were expressed in mPa·s.

### 2.9 Determination of Color Properties

The color values of the yogurt samples were determined using the technique outlined by Islam et al. (2014). A colorimeter BCM‐200 was used to determine the color values (L*, a*, and b*). The calibration of the meter was carried out using a white standard calibration plate. The L* value represents brightness from black (0) to white (100), the a* value indicates color ranging from red (+) to green (−), and the b* value indicates yellow (+) to blue (−).

### 2.10 Determination of Moisture Content

The oven dry method as reported by Islam et al. (2019) was used to calculate the percentage of moisture content in the yogurt samples. In short, 2 g of yogurt samples were dried at 105°C for 24 h in the oven. The formula used to compute the percentage moisture content is as follows:

$$\%\mathrm{MC}=\frac{\left(\;W1–W2\right)}{\;W1}\times 100$$

where W_1_ = initial weight of sample; W_2_ = weight of the dried sample.

### 2.11 Microbiological Analysis

The microbiological analysis of the yogurt samples was conducted using the method outlined by Min et al. (2022), with minor adjustments. All glassware, including petri dishes, pipettes, test tubes, flasks, and bottles, were sterilized in a hot air oven for 2 h, while distilled water and pipette tips were sterilized by autoclaving at 121°C for 15 min. To prepare the inoculums, each sample was serially diluted in sterile distilled water. Aliquots from each dilution were cultured on Nutrient Agar plates, and the total bacterial count was determined using the spread plate technique.

For media preparation, distilled water was combined with 3.75 g of agar powder and 2.5 g of agar nutrient media (Soybean Casein Digest Medium‐Trypton Soya Broth) to prepare a total volume of 200 mL of media solution, with a total of 350 mL prepared overall. The mixture was brought to a boil, then autoclaved at 121°C for 15 min to sterilize it. After cooling, 15–20 mL of the media was poured aseptically into each petri dish using a sterile pipette.

Serial dilutions were prepared by first sterilizing and labeling 40 test tubes approximately from 10^−1^ to 10^−8^ calibration. Each tube was filled with 9 mL of sterile distilled water. To create a 10^−8^ dilution, 1 mL of the sample was pipetted into the first tube labeled 10^−1^, and then 1 mL was transferred from each tube into the next sequentially, following the serial dilution process. Once the media had set, 0.1 mL of each diluted sample was plated onto sterile nutrient agar plates using a micropipette. The plates were then incubated at 37°C for 48 h to allow colony development.

### 2.12 Analysis of Microstructure

The yogurt samples were examined microscopically using scanning electron microscopy (SEM). Small slices of the internal part of the freeze‐dried samples were prepared, and an accelerating voltage of 10 kV was applied to observe the microstructure using a SEM (model SU1510, Hitachi High‐Tech, Tokyo, Japan), following the methods described by Congying et al. (2024). After 1 week of storage, the samples were freeze‐dried and then analyzed for their microstructural characteristics.

### 2.13 Analysis of the Sensory Quality

The sensory evaluation was conducted according to the methodology outlined by Ahiduzzaman et al. (2024). Thirty semi‐trained panelists, comprising teachers and students from the Department of Food Engineering and Technology at HSTU, participated in the analysis. The panelists had prior experience with sensory testing. They assessed the yogurt samples using a nine‐point hedonic scale to rate attributes such as color, texture, taste, flavor, mouthfeel, appearance, and overall acceptability (where 1 indicated dislike extremely, 9 indicated like extremely, and 5 represented neither like nor dislike). The coded yogurt samples, along with drinkable water for palate cleansing, were provided to the panelists, who were instructed to rinse their mouths after tasting each sample. Additionally, the panelists were encouraged to share their opinions and suggestions regarding the flavor, mouthfeel, and sensory texture of the samples.

### 2.14 Statistical Analysis

All experiments were conducted in triplicate to ensure reliability, and the results were expressed as the mean value accompanied by the standard deviation (mean ± SD). Statistical significance was considered at *p* < 0.05, as determined through Tukey's Honest Significant Difference (HSD) test. All statistical analyses were performed using R statistical software (R language version 3.3.4, R Core Team) through R Studio (RStudio version 1.1.423). Package “agricolae” was used for analysis of variance (ANOVA). For plotting the graphs, OriginPro software was used (OriginPro 2018b, OriginLab Corporation, USA) (Islam 2022).

## 3 Results and Discussion

### 3.1 The Physicochemical Properties of the Yogurt Samples

The physicochemical characteristics of yogurt samples, specifically moisture content, total solids, and color values, are summarized in Table 1. These parameters exhibited notable variations when rice milk and coconut milk were used as substitutes for cow milk in differing proportions.

**TABLE 1:** Physicochemical properties of the yogurt.

Properties	Storage days	Yogurt samples				
		S_1_	S_2_	S_3_	S_4_	S_5_
MC (%)	Day‐1	78.53 ± 0.351^Aa^	72.70 ± 0.265^Ad^	78.08 ± 0.225^Aa^	76.20 ± 0.200^Ac^	77.10 ± 0.361^Ab^
	Day‐7	75.27 ± 0.306^Bb^	70.17 ± 0.208^Bd^	75.97 ± 0.252^Ba^	74.10 ± 0.361^Bc^	75.03 ± 0.252^Bb^
L*	Day‐1	70.22 ± 0.202^Ac^	75.27 ± 0.379^Aa^	62.43 ± 0.404^Ae^	74.07 ± 0.208^Ab^	69.23 ± 0.252^Ad^
	Day‐7	64.07 ± 0.306^Bb^	70.17 ± 0.473^Ba^	54.13 ± 0.153^Bd^	70.00 ± 0.200^Ba^	60.30 ± 0.265^Bc^
a*	Day‐1	1.53 ± 0.025^Ac^	1.12 ± 0.020^Be^	1.93 ± 0.035^Aa^	1.22 ± 0.012^Ad^	1.73 ± 0.026^Bb^
	Day‐7	1.34 ± 0.035^Bc^	1.16 ± 0.010^Ad^	1.43 ± 0.020^Bb^	1.12 ± 0.029^Bd^	1.82 ± 0.044^Aa^
b*	Day‐1	10.52 ± 0.021^Ac^	9.71 ± 0.036^Ad^	11.13 ± 0.026^Aa^	9.32 ± 0.047^Ae^	10.73 ± 0.057^Ab^
	Day‐7	8.74 ± 0.045^Bb^	8.26 ± 0.038^Bc^	7.73 ± 0.026^Bd^	9.23 ± 0.035^Aa^	8.24 ± 0.030^Bc^

*Note:* Data represented as means ± standard deviation of three replicates. Different small superscript letters indicate significant differences among different samples in same storage days, while different capital superscript letters indicate significant differences among the storage days at (*p* < 0.05).

Abbreviations: S1, cow milk (control); S2, cow milk + SMP; S3, rice milk + SMP; S4, coconut milk + SMP; S5, rice coconut blend + SMP.

Both total solids and moisture content significantly impact the texture of yogurt, with higher total solids and lower moisture levels contributing to a firmer and more consistent texture, resulting in an acceptable mouthfeel (Priya 2016). On Day 1, the moisture content of the samples ranged from 78.53% ± 0.351% to 72.70% ± 0.265%, while by Day 7, it decreased to between 75.97% ± 0.252% and 70.17% ± 0.208%. Specifically, Sample S1 demonstrated the highest moisture content on Day 1; however, by Day 7, Sample S3 (predominantly rice milk) exhibited the highest moisture content. Sample S2 consistently showed the lowest moisture content on both days. Notably, these results were slightly below the 80.10%–85.23% moisture range reported for coconut‐based yogurt by Ndife (2014). On Day 1, moisture content values of S1 and S3 were analogous, whereas S2, S4, and S5 differed significantly (*p* < 0.05). By Day 7, S1 and S5 showed no significant difference. The moisture in all samples decreased over the storage period.

Color is a critical indicator of consumer preference and quality in the food industry, closely linked to flavor, taste, nutrition, and pigments (Pathare et al. 2012). L*, a*, and b* color values were recorded in Table 1. Sample S2, containing the highest total solids, had the highest L* value (75.27 ± 0.379 on Day 1 and 70.17 ± 0.473 on Day 7), whereas Sample S3 (rice milk) had the lowest L* value (62.43 ± 0.404 on Day 1 and 54.13 ± 0.153 on Day 7). Comparable L* values were reported by Akeem et al. (2018) for yogurts with high coconut milk content. On Day 1, significant differences in L*, a*, and b* values were observed (*p* < 0.05). By Day 7, no significant differences in a* and b* values were detected between Samples S2 and S4, illustrating consistency in color over the storage period.

**FIGURE 3:**
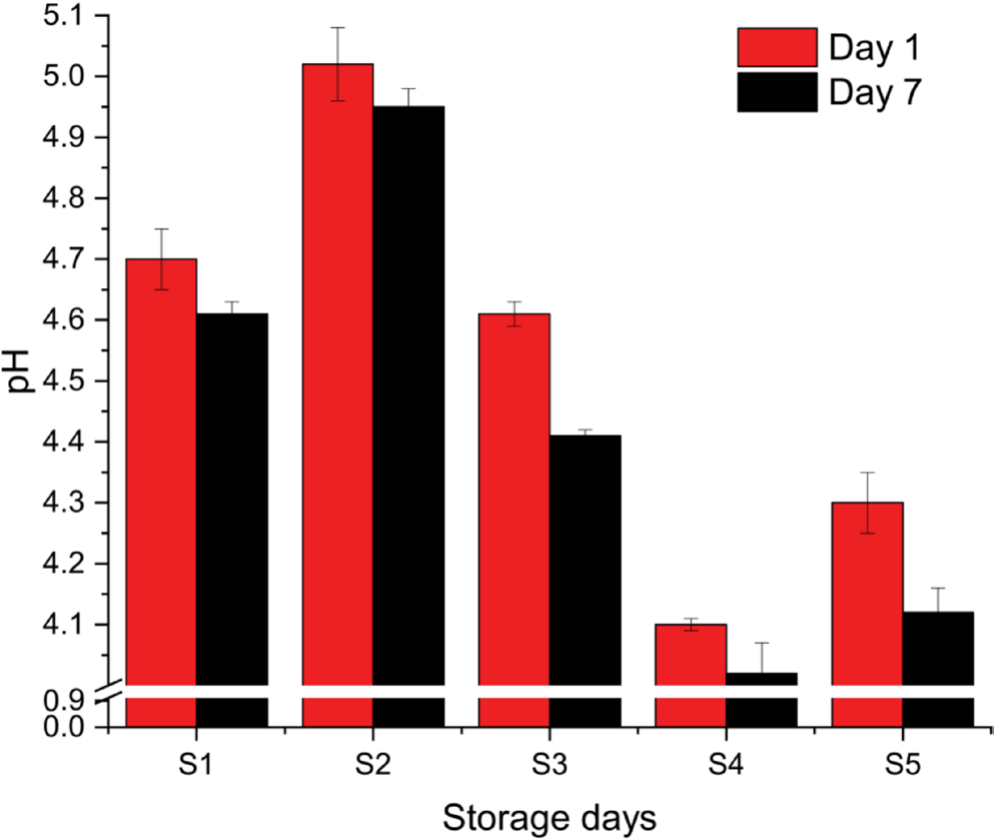
pH values of five different yogurt samples. Different small superscript letters indicate significant differences among different samples in same storage days, while different capital superscript letters indicate significant differences among the storage days at (*p* < 0.05). S1, cow milk (control); S2, cow milk + SMP; S3, rice milk + SMP; S4, coconut milk + SMP; S5, rice coconut blend + SMP.

Initially, the pH values for the yogurt samples ranged from 4.10 to 5.02 on Day 1 and decreased to a range of 4.02 to 4.95 by Day 7 (Figure 3). Sample S2 demonstrated the highest pH values at both storage intervals (5.02 ± 0.021 on Day 1 and 4.95 ± 0.044 on Day 7) and exhibited no significant change over the storage duration (*p* < 0.05). In contrast, pH values for all other samples significantly decreased by Day 7. Across all samples within each storage period, significant differences in pH were observed (*p* < 0.05).

**FIGURE 4:**
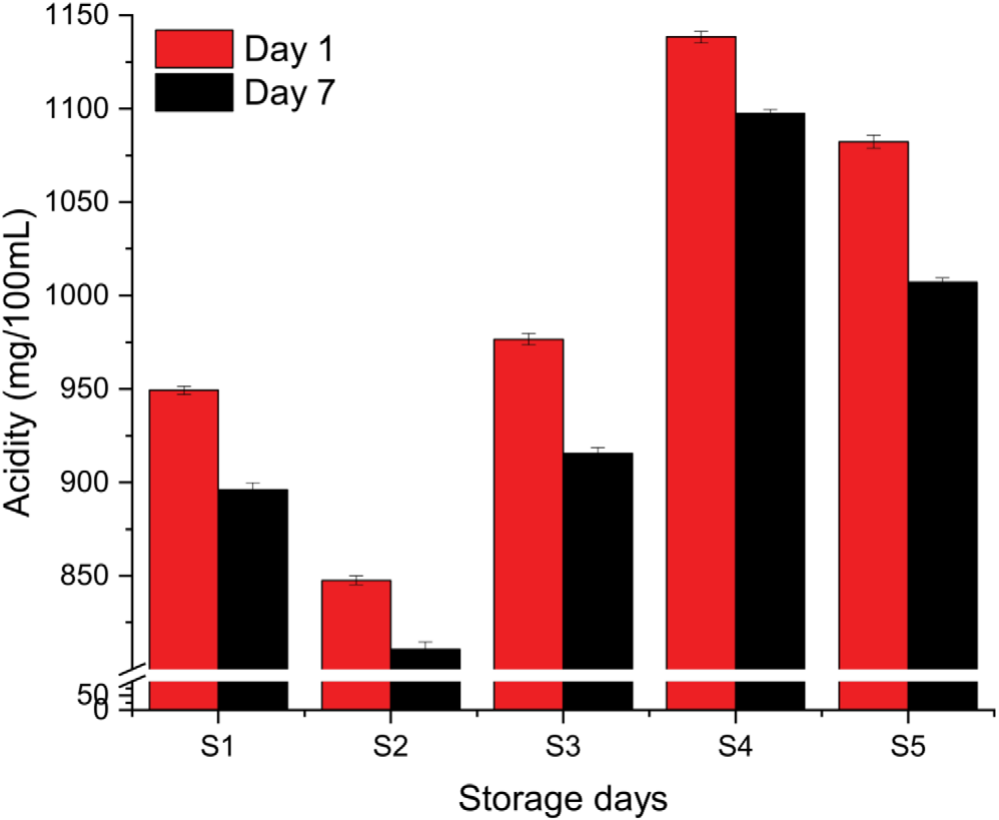
Acidity values of five different yogurt samples. Different small superscript letters indicate significant differences among different samples in the same storage days, while different capital superscript letters indicate significant differences among the storage days at (*p* < 0.05). S1, cow milk (control); S2, cow milk + SMP; S3, rice milk + SMP; S4, coconut milk + SMP; S5, rice coconut blend + SMP.

As anticipated, the acidity showed an inverse relationship with pH. All five yogurt samples exhibited significant differences in acidity (*p* < 0.05) at both Day 1 and Day 7 (Figure 4). Over time, acidity increased as pH decreased, indicating continuing fermentation. Sample S4, containing the highest proportion of coconut milk, showed the highest acidity values (1097.67 ± 7.51 on Day 1 and 1138.33 ± 9.07 on Day 7) and correspondingly the lowest pH values (4.10 ± 0.021 on Day 1 and 4.02 ± 0.031 on Day 7). Sample S5, which included 50% coconut milk, displayed higher acidity than sample S3 (rice milk yogurt) due to its coconut milk content. Conversely, Sample S2, composed solely of normal milk and milk powder, had the lowest acidity (810.67 ± 9.02 on Day 1 and 847.67 ± 7.51 on Day 7), aligning with its higher pH values.

These findings are consistent with previous studies. Fawzi et al. (2022) reported a similar trend of decreasing pH and increasing acidity over time in rice milk yogurt. The observed low pH in high coconut milk yogurts agrees with the results of Akeem et al. (2018) and Priya (2016), confirming that coconut milk formulations can lead to enhanced acidity due to its compositional properties. Therefore, the formulation of yogurt with varying coconut and rice milk contents significantly influences the acidification process during storage.

**FIGURE 5:**
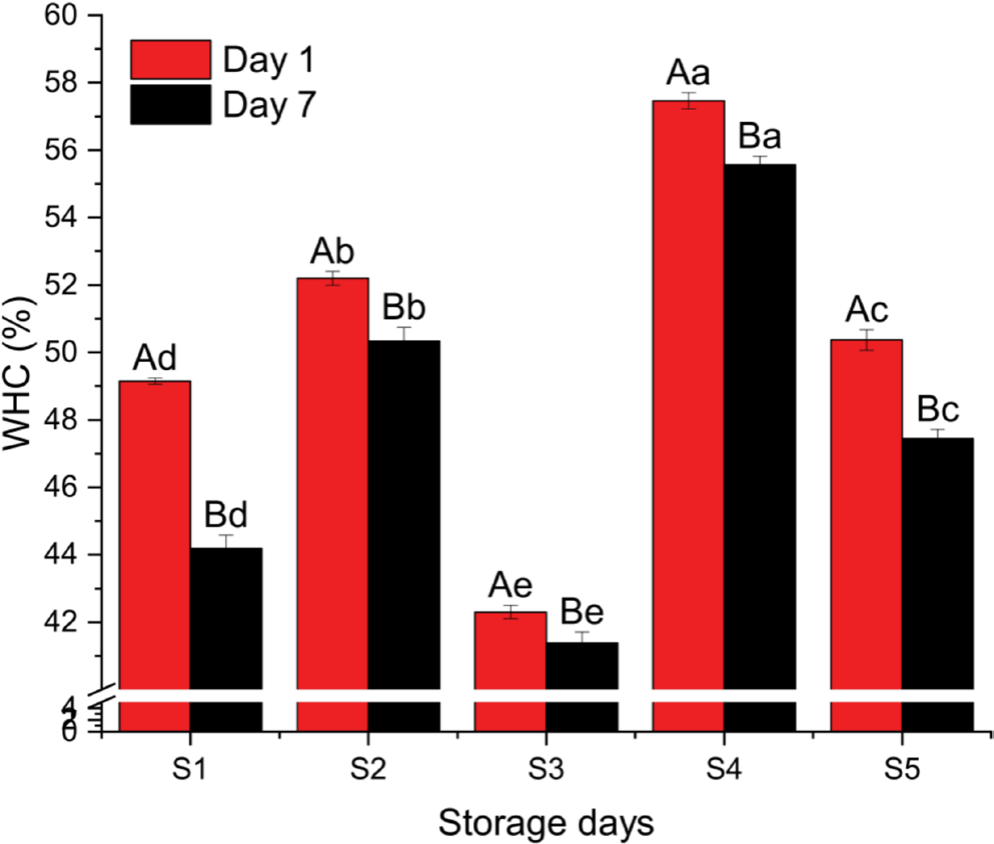
Water holding capacity (WHC) of different yogurt samples. Different small superscript letters indicate significant differences among different samples in same storage days, while different capital superscript letters indicate significant differences among the storage days at (*p* < 0.05). S1, cow milk (control); S2, cow milk + SMP; S3, rice milk + SMP; S4, coconut milk + SMP; S5, rice coconut blend + SMP.

The water holding capacity (WHC), syneresis, total soluble solids (TSS), and viscosity values of the yogurt samples were examined, with results depicted in Figure 5 through 8. These figures illustrate the impact of different milk formulations on key textural and compositional characteristics of yogurt over storage time.

Figure 5 highlights the variations in WHC among the samples across the storage period. The interaction between protein and water is crucial for yogurt's flavor and texture, as WHC is predominantly determined by the water retention within the protein matrix (Landge 2009). An increased WHC suggests a more hydrated protein network. Sample S4, with a high percentage of coconut milk, exhibited the highest WHC (57.46 ± 0.174 on Day 1 and 55.57 ± 0.306 on Day 7), indicating superior water retention. Conversely, Sample S3, which consisted primarily of rice milk, showed the lowest WHC throughout the storage period (42.3 ± 0.361 on Day 1 and 41.40 ± 0.335 on Day 7). Significant differences in WHC were observed among all samples both at the onset and conclusion of storage (*p* < 0.05).

A declining trend in WHC was noted for all samples during storage, implying a weakening gel structure and potential for phase separation or whey separation (Singh and Muthukun 2008). It is worth noting that stabilizers can enhance WHC by binding water more effectively and improving overall texture (Thaiudom and Goff 2003).

Syneresis is generally considered a flaw in high‐quality yogurt and tends to be more pronounced in the absence of stabilizers within the yogurt matrix (Mohsin et al. 2019). Over time, syneresis can negatively affect the texture and structural integrity of yogurt. Previous research indicates that low‐fat yogurts typically exhibit higher syneresis levels compared to high‐fat yogurts (Staff, M 1998). A lower syneresis value is desirable, as it enhances consumer acceptability and indicates better gel stability.

**FIGURE 6:**
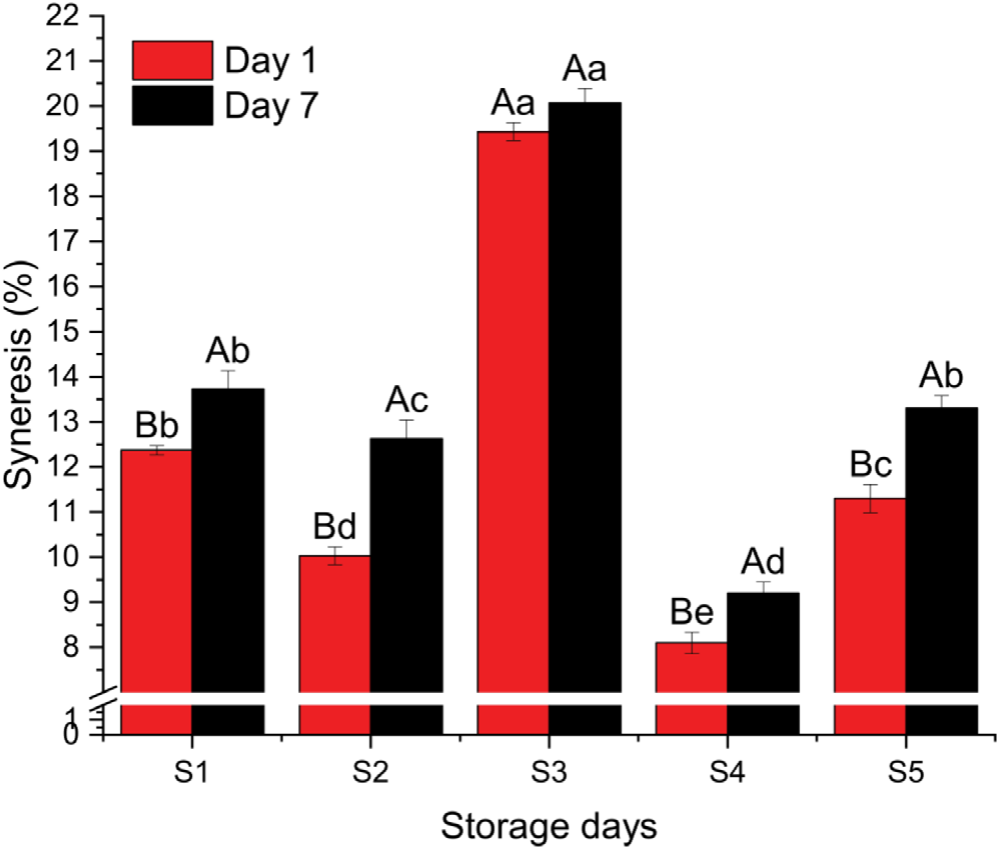
Syneresis of different yogurt samples. Different small superscript letters indicate significant differences among different samples in same storage days, while different capital superscript letters indicate significant differences among the storage days at (*p* < 0.05). S1, cow milk (control); S2, cow milk + SMP; S3, rice milk + SMP; S4, coconut milk + SMP; S5, rice coconut blend + SMP.

Figure 6 illustrates the syneresis results for the different yogurt samples. The syneresis percentage ranged from 8.10 ± 0.173% to 19.43 ± 0.404% on Day 1, and from 9.20 ± 0.265% to 20.07 ± 0.404% on Day 7. The highest syneresis was observed in Sample S3, which contained the highest proportion of rice milk. Conversely, Sample S4, enriched with a higher amount of coconut milk, exhibited the lowest syneresis, likely due to its higher fat content.

No significant differences in syneresis were observed between Sample S1 and S5 at Day 7 (*p* < 0.05), nor between the two storage periods for Sample S3. Overall, syneresis increased throughout storage in all samples, indicating a weakening of the gel network over time. The lower syneresis observed in coconut milk‐based yogurt (Sample S4) may be attributed to the higher fat content, which helps stabilize the gel and prevent whey separation.

**FIGURE 7:**
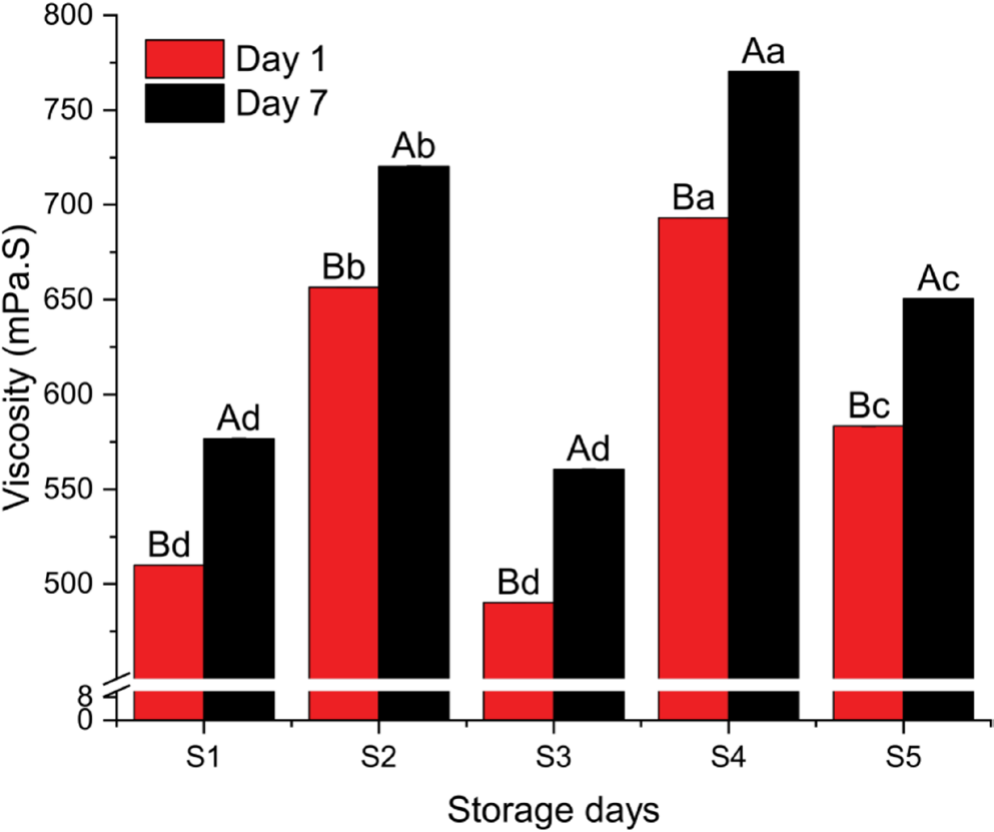
Viscosity of different yogurt samples. Different small superscript letters indicate significant differences among different samples in same storage days, while different capital superscript letters indicate significant differences among the storage days at (*p* < 0.05). S1, cow milk (control); S2, cow milk + SMP; S3, rice milk + SMP; S4, coconut milk + SMP; S5, rice coconut blend + SMP.

Figure 7 illustrates the variation in viscosity among the different yogurt samples, with error bars indicating the standard deviations. The viscosity values on Day 1 ranged from 490.00 ± 20 mPa·s (Sample S3) to 693.33 ± 15.28 mPa·s (Sample S4). By Day 7, the viscosity increased in all samples, with values ranging from 560.00 ± 26.46 mPa·s (S3) to 770.00 ± 20 mPa·s (S4). Specifically, the viscosity of Sample S1 was 510.00 ± 10 mPa·s at Day 1 and 576.67 ± 15.28 mPa·s at Day 7; for S2, it was 656.67 ± 11.55 and 720.00 ± 10, respectively; for S3, it was 490.00 ± 20 and 560.00 ± 26.46; for S4, 693.33 ± 15.28 and 770.00 ± 20; and for S5, 583.33 ± 23.09 and 650.00 ± 17.32.

Statistical analysis revealed that the viscosity values of S1 and S3 did not differ significantly between Day 1 and Day 7 (*p* < 0.05). However, all other samples showed significant increases in viscosity over the storage period. The highest viscosity was observed in Sample S4, likely due to its higher coconut milk content, consistent with the findings of Imele and Atemnkeng (2001), who noted that viscosity increases with higher coconut milk proportions. Conversely, Sample S3, containing mostly rice milk, exhibited the lowest viscosity. Overall, the viscosity trend indicates that rice milk‐based yogurt tends to decrease in viscosity over time, whereas coconut milk‐based yogurt demonstrates an increasing viscosity during storage, possibly due to structural changes and fat content interactions.

**FIGURE 8:**
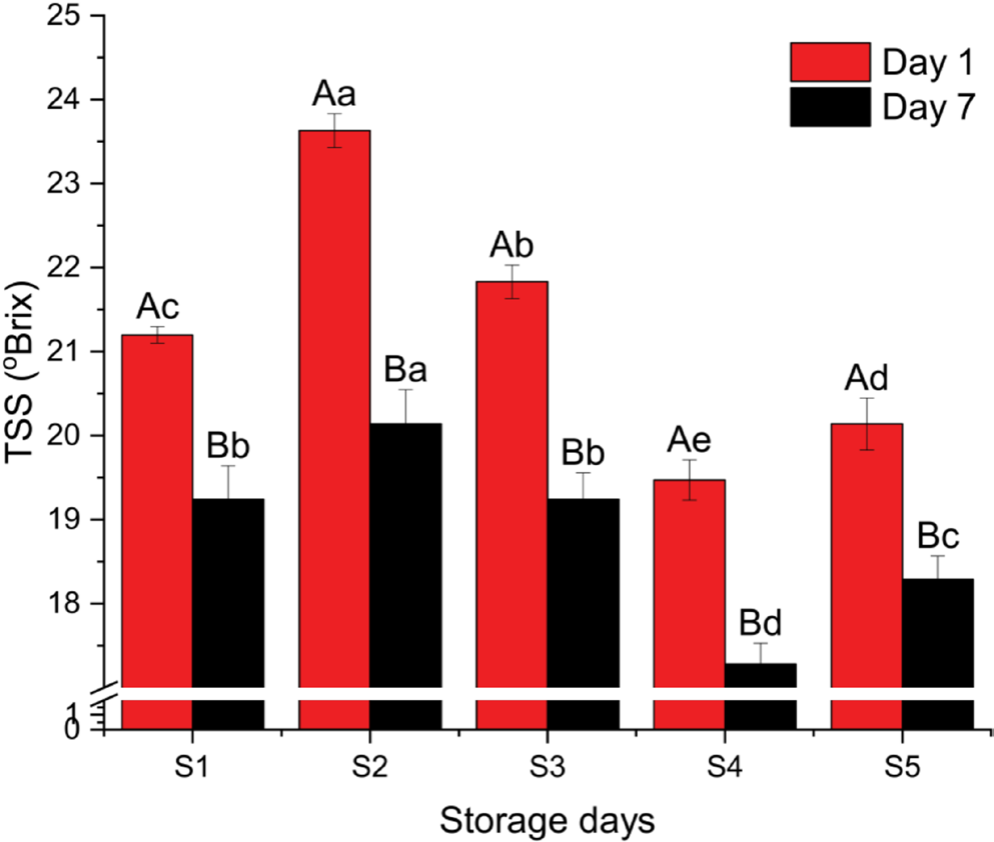
TSS of different yogurt samples. Different small superscript letters indicate significant differences among different samples in same storage days, while different capital superscript letters indicate significant differences among the storage days at (*p* < 0.05). S1, cow milk (control); S2, cow milk + SMP; S3, rice milk + SMP; S4, coconut milk + SMP; S5, rice coconut blend + SMP.

Figure 8 depicts the total soluble solids (TSS) values of the five yogurt samples, with error bars indicating the standard deviations. On Day 1, TSS ranged from 19.47% ± 0.306% (Sample S4) to 23.63% ± 0.153% (Sample S2). By Day 7, the values decreased to a range of 17.37% ± 0.321% (S4) to 20.30% ± 0.173% (S2). The highest TSS was observed in Sample S2, while Sample S4 showed the lowest. Statistically, the TSS values of S1 and S3 did not differ significantly at Day 7 (*p* < 0.05), whereas all other samples showed significant differences between Day 1 and Day 7.

The TSS value of Sample S4 was slightly higher than reported by Shristi and Gita (2019), which could be attributed to variations in coconut milk preparation and yogurt manufacturing procedures. A clear decreasing trend in TSS was observed during storage, consistent with findings by Dzigbordi et al. (2013), which attributed this reduction to increases in acidity levels over time. The increase in acidity during storage likely accelerates the breakdown of soluble solids, contributing to the observed decline.

Total number of bacteria present in S_2_, S_3_, S_4_ and S_5_ samples was calculated and compared with control sample S_1_. Total plate count of yogurt is presented in the Table 2 below:

**TABLE 2:** Total microbial count in five different yogurt samples.

Storage periods	Samples				
	S_1_	S_2_	S_3_	S_4_	S_5_
Day1 (log CFU/g)	10.10 ± 0.142^Aa^	10.27 ± 0.093^Aa^	9.08 ± 0.115^Ab^	7.81 ± 0.191^Ad^	8.43 ± 0.08^Ac^
Day7 (log CFU/g)	10.23 ± 0.069^Aa^	10.38 ± 0.126^Aa^	9.17 ± 0.104^Ab^	7.94 ± 0.251^Ad^	8.63 ± 0.121^Ac^

*Note:* Data represented as means ± standard deviation of three replicates. Different small superscript letters indicate significant differences among different samples in same storage days, while different capital superscript letters indicate significant differences among the storage days at (*p* < 0.05).

Abbreviations: S1, cow milk (control); S2, cow milk + SMP; S3, rice milk + SMP, S4, coconut milk + SMP; S5, rice coconut blend + SMP.

Table 2 presents the total plate counts of the five yogurt samples. Yogurt containing *Lactobacillus bulgaricus* and *Streptococcus thermophilus* is known to promote human health, with an optimal level of approximately 10^7^ CFU/mL (Rahman et al. 2024). The total bacterial counts in the samples ranged from 10.27 ± 0.093 log CFU/g to 7.81 ± 0.191 log CFU/g on Day 1, and from 10.38 ± 0.126 log CFU/g to 7.94 ± 0.251 log CFU/g on Day 7. Significant differences in bacterial counts were observed among Samples S3, S4, and S5 at both Day 1 and Day 7 (*p* < 0.05), whereas no significant differences were found between S1 and S2.

Interestingly, the total microbial count remained relatively stable within each sample across the two storage periods. The highest microbial count was recorded in S2, containing the added SMP, which likely provided a more favorable environment for probiotic bacteria. Conversely, the counts in S3, S4, and S5 decreased compared to the control (S1), possibly because many probiotic strains are originally isolated from dairy sources, and the lower nutrient availability, presence of anti‐nutritional factors, and the pH of vegan milks may hinder their growth and survival (Rasika et al. 2021). Nandakumar et al. (2022) observed a decline in microbial counts beyond Day 2, whereas we noted an increase at Day 7, which could be attributed to the consistent use of 5% bacterial culture. Further studies examining storage periods beyond 7 days are necessary to better understand the long‐term viability and stability of probiotic bacteria in plant‐based yogurts.

### 3.2 Sensory Evaluation

**FIGURE 9:**
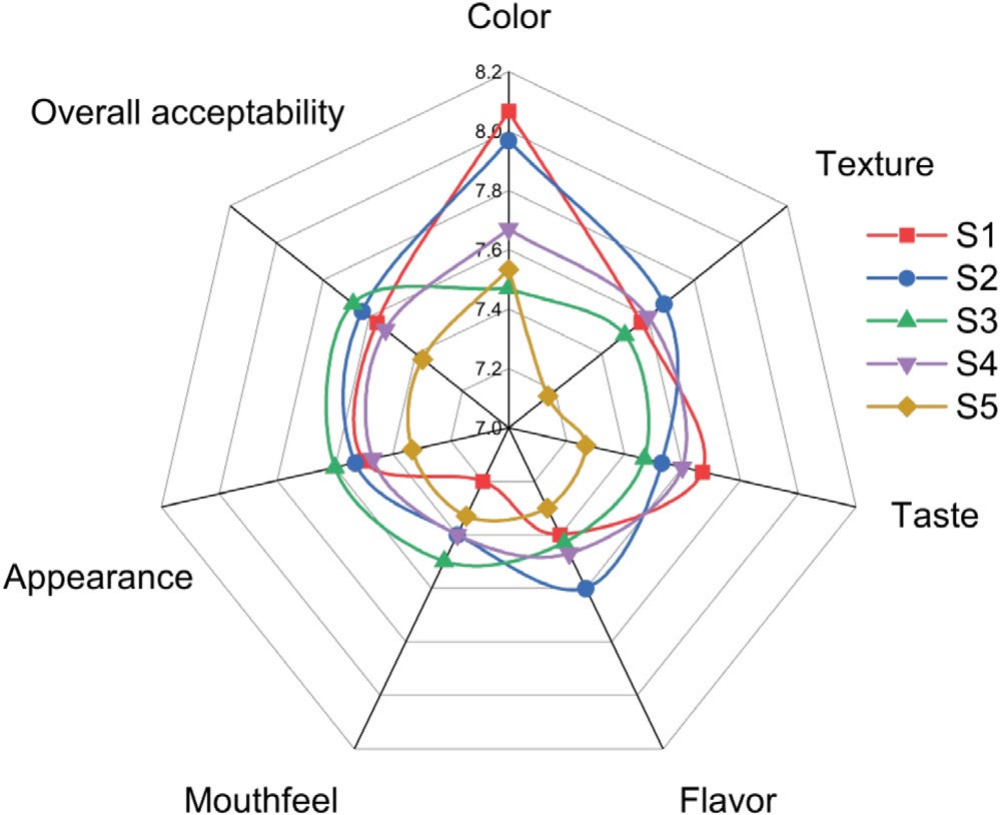
Sensory evaluation of yogurt samples. S1, cow milk (control); S2, cow milk + SMP; S3, rice milk + SMP; S4, coconut milk + SMP; S5, rice coconut blend + SMP.

Figure 9 shows the mean scores for color, texture, taste, flavor, mouthfeel, appearance, and overall acceptability of the yogurt samples. The organoleptic evaluation was conducted at Day 1 of storage, assessing the impact of rice milk and coconut milk on sensory properties. A panel of approximately 30 semi‐trained participants rated each parameter, and a one‐way ANOVA was performed to identify any significant differences between samples at the significance level of (*p* < 0.05).

The results indicated that there were no substantial differences in sensory attributes among the samples. Specifically, Samples S1 and S2, representing the control and the sample with added SMP, did not differ significantly from those produced with rice and coconut milk in any of the sensory categories. These findings align with those of Akeem et al. (2018), who reported no significant variations in taste or mouthfeel between cow‐coconut milk yogurts.

In terms of specific attributes, the control (S1) scored the highest for color and taste, while S2 excelled in texture and flavor. Conversely, Sample S3 (rice milk yogurt) received the highest scores for mouthfeel, appearance, and overall acceptability, suggesting good consumer acceptance. The scores for coconut milk yogurt (S4) for texture, flavor, and mouthfeel exceeded those of the control, with taste, appearance, and overall acceptability scoring similarly. Additionally, S4 outperformed S3 in color, taste, and flavor scores.

Overall, the sensory evaluation demonstrated that both rice milk and coconut milk can produce yogurt with acceptable organoleptic qualities, indicating their potential as viable alternatives to traditional dairy yogurt.

### 3.3 Microstructure of Yogurt Samples

**FIGURE 10:**
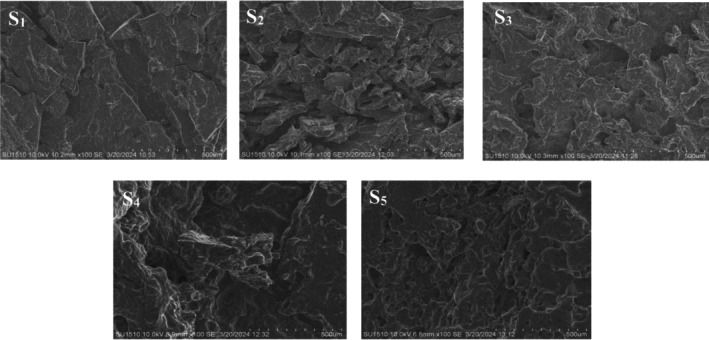
Scanning Electron Microscopic photos of yogurt samples. S1, cow milk (control); S2, cow milk + SMP; S3, rice milk + SMP; S4, coconut milk + SMP; S5, rice coconut blend + SMP.

Figure 10 illustrates the microstructure of the yogurt samples after 1 week of storage. The micrographs reveal that the yogurt's microstructure comprised numerous interconnecting chains of asymmetrically arranged casein micelles, with fat globules entrapped within the protein network. Each sample exhibited a unique microstructure, with S2, S3, S4, and S5 displaying distinct features compared to the control (S1). These observations are consistent with the findings of Kamal‐Eldin et al. (2020). Specifically, Sample S1 showed thicker casein chains, smaller and fewer unoccupied spaces, and larger casein micelle clusters, indicating a dense and well‐developed protein network. Sample S2, which contained more coconut milk, displayed larger and more numerous empty spaces, along with coarser structures and a more robust protein matrix. When rice milk was used in S3, the casein network appeared smoother, with smaller micelle clusters, thinner chains, and fewer empty spaces, suggesting a more uniform and less coarse microstructure.

Sample S4 exhibited larger, but fewer, empty spaces, along with coarser and thicker chains, indicating a coarser overall network. Lastly, S5 demonstrated a more orderly and evenly distributed casein network, with smaller, more numerous empty spaces and smaller micelle clusters overall. Notably, S2 and S4, which showed higher water‐holding capacities in physicochemical analyses, also exhibited a granular or coarser microstructure, likely due to the denser protein matrix and fat globule entrapment.

The observed differences in physicochemical and sensory properties among the yogurt samples can be attributed to variations in their formulation. Coconut milk, being rich in lipids, likely contributed to higher fat content and improved water‐holding capacity, which may enhance the overall nutritional profile. Rice milk, primarily composed of carbohydrates, influences the carbohydrate content and gel formation during fermentation. The addition of skim milk powder increased protein levels and solids content, thereby improving texture and enhancing probiotic viability. These formulation differences not only affected the physicochemical characteristics but also support the concept of creating nutritionally tailored plant‐based yogurts that can serve as functional alternatives.

While this study primarily emphasizes physicochemical, microbiological, and sensory attributes, a detailed proximate and textural profile analysis, including parameters such as moisture, crude protein, fat, ash, crude fiber, and carbohydrate content, is necessary to thoroughly assess their nutritional profiles. Future research should incorporate comprehensive proximate nutrient profiling to better compare the nutritional benefits and contributions of rice milk, coconut milk, and traditional cow milk yogurts. Additionally, the functional and bioactive properties, including antioxidant activity, phenolic compounds, probiotic viability, bioaccessibility, lipid oxidation, phase separation, as well as probiotic viability over extended storage periods, are critical factors that warrant further investigation. Future studies should explore these aspects to comprehensively assess the health‐promoting potential of these plant‐based yogurt formulations.

## 4 Conclusion

This study highlights the potential of rice and coconut milk as effective bases for producing nutritious and consumer‐acceptable yogurts that rival traditional dairy yogurt in key physicochemical and microbiological attributes. Coconut milk yogurt exhibited superior physicochemical stability, featuring reduced syneresis and heightened acidity, alongside favorable sensory qualities, which establish it as a promising dairy alternative. Although slightly less robust in certain physicochemical aspects, rice milk yogurt maintained satisfactory quality and strong consumer acceptance, underscoring its viability as a dairy substitute.

These results support the potential of plant‐based yogurts to cater to individuals with lactose intolerance and dairy allergies, broadening their access to healthier dietary choices. Future investigations should focus on conducting detailed proximate analyses to better articulate the nutritional contributions of these formulations. Additionally, researching their functional and bioactive properties, such as antioxidant capacity, phenolic content, and probiotic viability, as well as studying the effects of stabilizers or thickeners, will be critical. Such efforts can enhance the microstructural stability and shelf life of these products, ultimately fostering the advancement of functional plant‐based dairy alternatives in the nutrition market.

## Author Contributions

**Md. Nahidul Islam:** conceptualization, methodology, validation, software, data curation, supervision, resources, writing – review and editing, writing – original draft, investigation.

## Ethics Statement

This study does not involve any human or animal testing.

## Conflicts of Interest

The authors declare no conflicts of interest.

## Data Availability

The data that support the findings of this study are available from the corresponding author upon reasonable request.
